# Curcumin activates DNA repair pathway in bone marrow to improve carboplatin-induced myelosuppression

**DOI:** 10.1038/s41598-017-16436-9

**Published:** 2017-12-18

**Authors:** Xiao Chen, Jigang Wang, Zhongping Fu, Bo Zhu, Jie Wang, Shengwen Guan, Zichun Hua

**Affiliations:** 1 The State Key Laboratory of Pharmaceutical Biotechnology, School of Life Sciences, Nanjing University, Nanjing, 210023 China; 2 State Key Laboratory of Quality Research in Chinese Medicines, Macau University of Science and Technology, Macau, 999078 China; 3 Department of Biological Science, National University of Singapore, Singapore, 117543 Singapore; 4 Changzhou High-Tech Research Institute of Nanjing University and Jiangsu TargetPharma Laboratories Inc., Changzhou, 213164 China; 5 Shenzhen Research Institute of Nanjing University, Shenzhen, 518057 China

## Abstract

Carboplatin, a second-generation platinum agent, has been used as a cancer therapy for decades and exhibits strong anti-tumor activity. However, the wide application of carboplatin is largely limited due to its side effects, especially myelosuppression. Here, we combined carboplatin with curcumin, a natural product that improves tumor-induced anemia, for the treatment of fibrosarcoma to improve the side effects of carboplatin. We first examined the synergistic and attenuated effects of the two agents in a T241-bearing mouse model. The combination therapy caused no obvious synergistic effect, but curcumin significantly improved the survival rate of carboplatin-treated mice. Histologic analysis of the kidney and bone marrow revealed that curcumin improved carboplatin-induced myelosuppression but did not affect the kidney. To determine the mechanism involved, we introduced a probe derived from curcumin to identify its targets in bone marrow cells and the results provided us a clue that curcumin might affect the DNA repair pathway. Western blot analysis revealed that curcumin up-regulated BRCA1, BRCA2 and ERCC1 expression in bone marrow. In conclusion, curcumin attenuates carboplatin-induced myelosuppression by activating the DNA repair pathway in bone marrow cells.

## Introduction

Platinum-based drugs, the most commonly used chemotherapeutic agents, have been used for many years for the treatment of several types of cancer, including ovarian, cervical, head and neck, and non-small-cell lung cancer^1,2^. Although platinum-based drugs exhibit strong anticancer activity, their usage is largely limited due to their serious side effects^3^. For example, cisplatin, a first-generation platinum agent, causes nephrotoxicity, neurotoxicity and ototoxicity, and it shows a small advantage in prolonging survival compared to best supportive care alone^4^. Consequently, carboplatin, a second-generation platinum drug with fewer side effects, was developed. Distinguished with cisplatin, carboplatin rarely results in nephrotoxicity and peripheral neuropathy, with its major toxic effect being myelosuppression^5^.

As one of the most widely used chemotherapeutic agents, the mechanism of toxicity of carboplatin has been largely studied^3,6–8^. The major toxic effect of carboplatin is myelosuppression, which occurs in approximately 20–40% of patients treated with conventional does of carboplatin and in more than 90% of patients treated with high-dose carboplatin. The main symptoms of carboplatin-induced myelosuppression include thrombocytopenia and neutropenia. As a result, carboplatin-induced myelosuppression leads to anemia in patients^9,10^ and largely limits carboplatin’s therapeutic effect of prolonging life span of tumor-bearing patients. Therefore, attenuating the toxicity of carboplatin has become a hot target for cancer therapy^11,12^.

Curcumin, a natural polyphenol from turmeric, has been used for centuries to treat inflammation, tumor and other diseases^13^. Many reports have shown that curcumin exhibits significant antiproliferative and apoptotic effects in a variety of tumor cell lines and inhibits the formation of reactive oxygen species (ROS)^14–17^. Interestingly, the effect of curcumin on platinum-based drugs induced toxicity has been largely reported. Zhang *et al*. found that curcumin significantly increased tolerance of bone marrow cells to chemotherapy-induced toxicity^18^. Antunes *et al*. reported that curcumin could prevent cisplatin-induced clastogenesis by acting as a free radical scavenger^19^. In addition, in our previous study, curcumin improved defective hematopoiesis induced by tumor-derived VEGF in a tumor model^20^.

Considering curcumin’s anti-cancer activity and improvement in hematopoiesis, the combination of carboplatin and curcumin would likely be a good treatment option for cancer therapy. On one hand, as both agents exhibit anticancer activity, the combination may lead to improved therapeutic effects. On the other hand, because carboplatin induces myelosuppression while curcumin improves defective hematopoiesis, curcumin likely attenuates myelosuppression induced by carboplatin. In this study, we will examine the effects of the combined drug therapy on tumor growth, kidney and bone marrow in T241-bearing mice and explore the mechanisms involved with a chemical proteomics approach.

## Results

### The combination of carboplatin and curcumin exhibits no synergistic effect but significantly improves the survival rate of tumor-bearing mice

**Figure 1:**
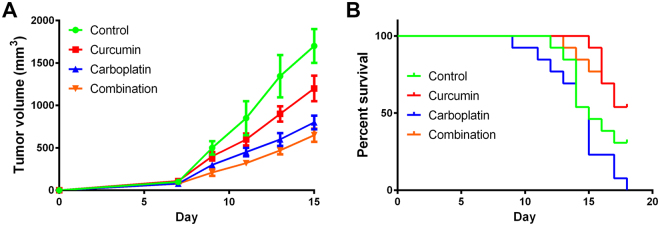
(**A**) Rates of Tumor growth in control-, curcumin-, carboplatin- and combination- treated groups (n = 8 – 10 mice/group). (**B**) Survival rates of control-, curcumin-, carboplatin- or combining agents- treated groups. The drug concentrations for *in vivo* experiments in the study are: control (0.1 mL 20% Tween-80 per mouse per day), curcumin (0.1 mL 5 mM curcumin per mouse per day), carboplatin (0.1 mL 5 mg/mL carboplatin per mouse per day) and combination (0.1 mL injection consisting of 5 mM curcumin and 5 mg/mL carboplatin per mouse per day).

Since both agents, carboplatin and curcumin, exhibit anticancer activity, we first examined the effects of the combination on tumor growth in a tumor-bearing mouse model. C57B6 mice were injected with T241 fibrosarcoma cells. Four groups of mice were treated with a series of intraperitoneal injections of 20% Tween-80 (control group), carboplatin alone, curcumin alone, or a combination of curcumin and carboplatin. Tumor growth was measured with calipers each day. Carboplatin and curcumin displayed no significant synergitic effect on the inhibition of tumor volume (Fig. 1A). Particularly, the inhibitory effects on tumor volume observed in animals treated with the combined drugs were stronger than that of control and curcumin group, and were similar to that of carboplatin group. Based on the survivorship curves, treatment with curcumin coupled with carboplatin remarkably promoted the survival rate of tumor-bearing mice compared to that of the carboplatin group (Fig. 1B). These results indicate that curcumin attenuates the side effects induced by carboplatin to promote survival.

### Curcumin has no effect on the kidney in tumor-bearing mice treated with carboplatin

**Figure 2:**
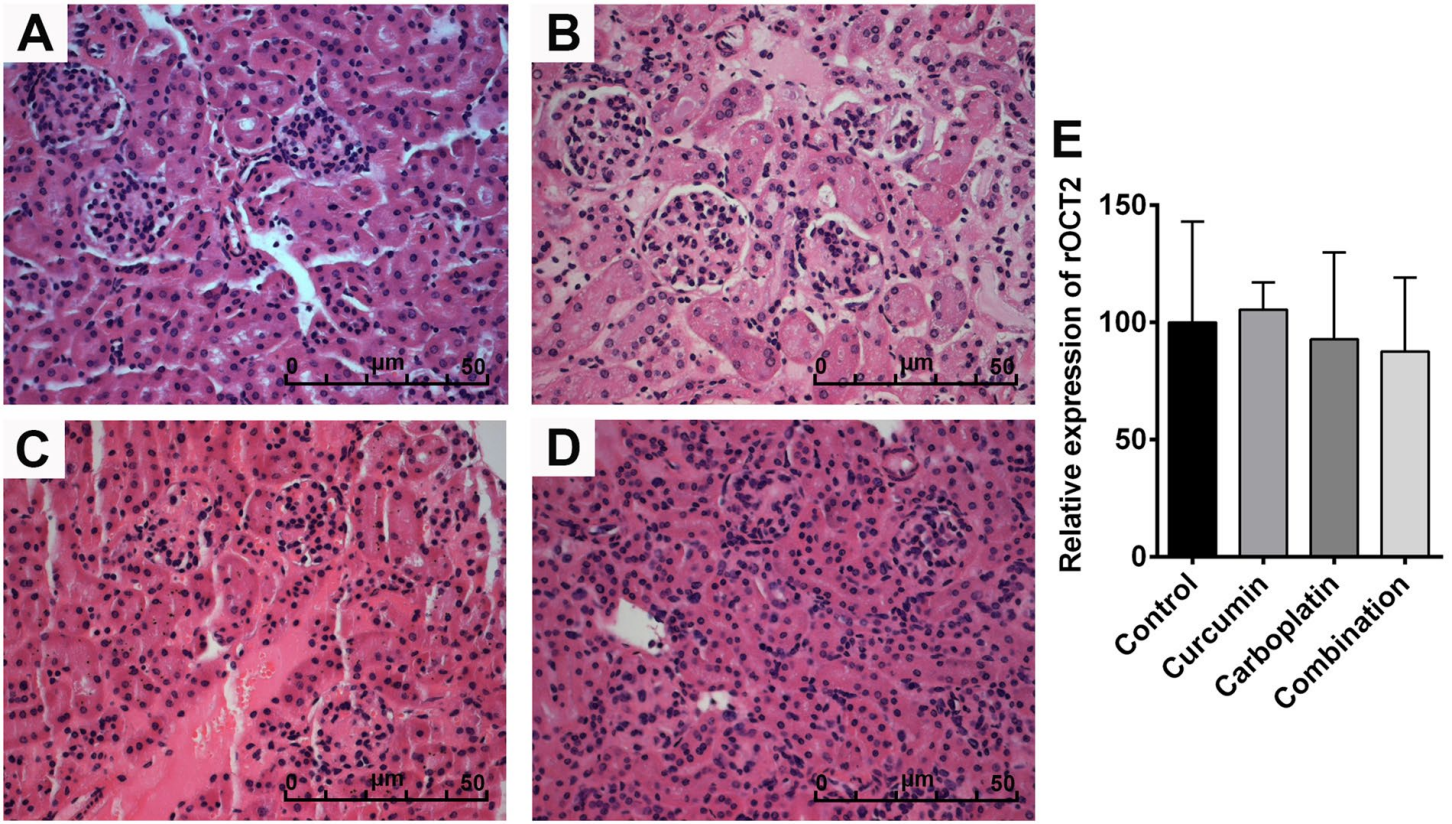
Kidney histology of (**A**) control-, (**B**) curcumin-, (**C**) carboplatin- and (**D**) combination-treated mice. (**E**) qPCR was used to examine rOCT2 expression in the kidneys of control-, curcumin-, carboplatin- and combination- treated mice.

Nephrotoxicity is associated with cisplatin treatment, and the kidney accumulates a higher effective concentration of cisplatin than any other organ after treatment with cisplatin^21,22^. This accumulation preferentially affects the terminal proximal tubule and the distal nephron and can cause either apoptosis or necrosis, depending on exposure time and concentration^21^. Previous reports showed that the nephrotoxicity of platinum-based drugs is related to the production of ROS. Considering curcumin’s inhibition of ROS formation, we inferred that curcumin might improve platinum-based drugs-induced nephrotoxicity. Therefore, we examined the effects of carboplatin or curcumin on the kidney in tumor-bearing mice. HE staining showed that carboplatin exhibited no nephrotoxicity, and curcumin did not affect the kidney (Fig. 2A–D). To further validate the results, qPCR was conducted to determine the expression of organic cation transporter 2 (rOCT2), a marker of nephrotoxicity^23–25^, in kidneys of tumor-bearing mice from different groups. Consistently, carboplatin or curcumin did not affect rOCT2 mRNA level (Fig. 2E), indicating that curcumin attenuates carboplatin-induced side effects not through improving nephrotoxicity.

### Curcumin attenuates myelosuppression induced by carboplatin

**Figure 3:**
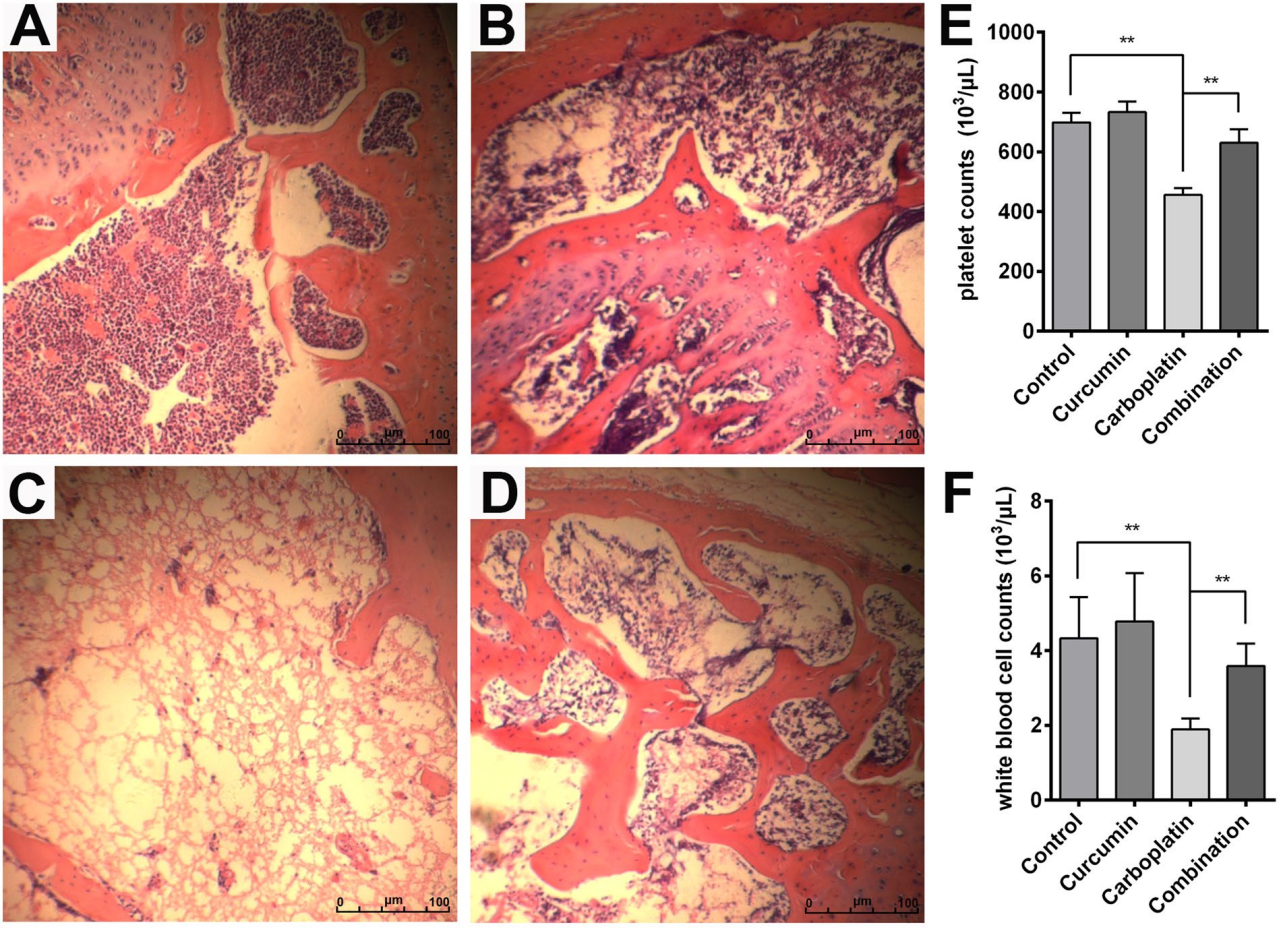
Bone marrow histology of (**A**) control-, (**B**) curcumin-, (**C**) carboplatin- and (**D**) combinations-treated mice. (**E**) Platelet counts in the peripheral blood of control-, curcumin-, carboplatin- and combination- treated mice. (**F**) White blood cell counts in the peripheral blood of control-, curcumin-, carboplatin- and combination- treated mice. **p < 0.01.

As myelosuppression is the major toxic effect of carboplatin, we next examined the effects of carboplatin or curcumin on the bone marrow. Thighbone HE sections showed that curcumin did not affect the marrow (Fig. 3A and B), while carboplatin caused serious damage to the marrow, with the disappearance of bone trabecula and fibrosis of hematopoietic cells (Fig. 3C). Treatment with curcumin significantly improved carboplatin-induced bone marrow damage, with a reappearance of bone trabecular and defibrillation of hematopoietic cells (Fig. 3D).

Since carboplatin and curcumin dramatically affect the marrow, we inferred that the two agents may also affect the hematopoietic function. Blood was analyzed with a hematology analyzer to validate our hypothesis. Carboplatin significantly decreased the platelet and white blood cell counts in treated animals, while the combination group recovered to the pretreatment levels (Fig. 3E and F). These findings reveal that carboplatin is harmful to hematopoietic function, and curcumin improves carboplatin-induced hematopoietic dysfunction, which is consistent with the HE staining results.

### Curcumin improves DNA damage induced by carboplatin in bone marrow cells

**Figure 4:**
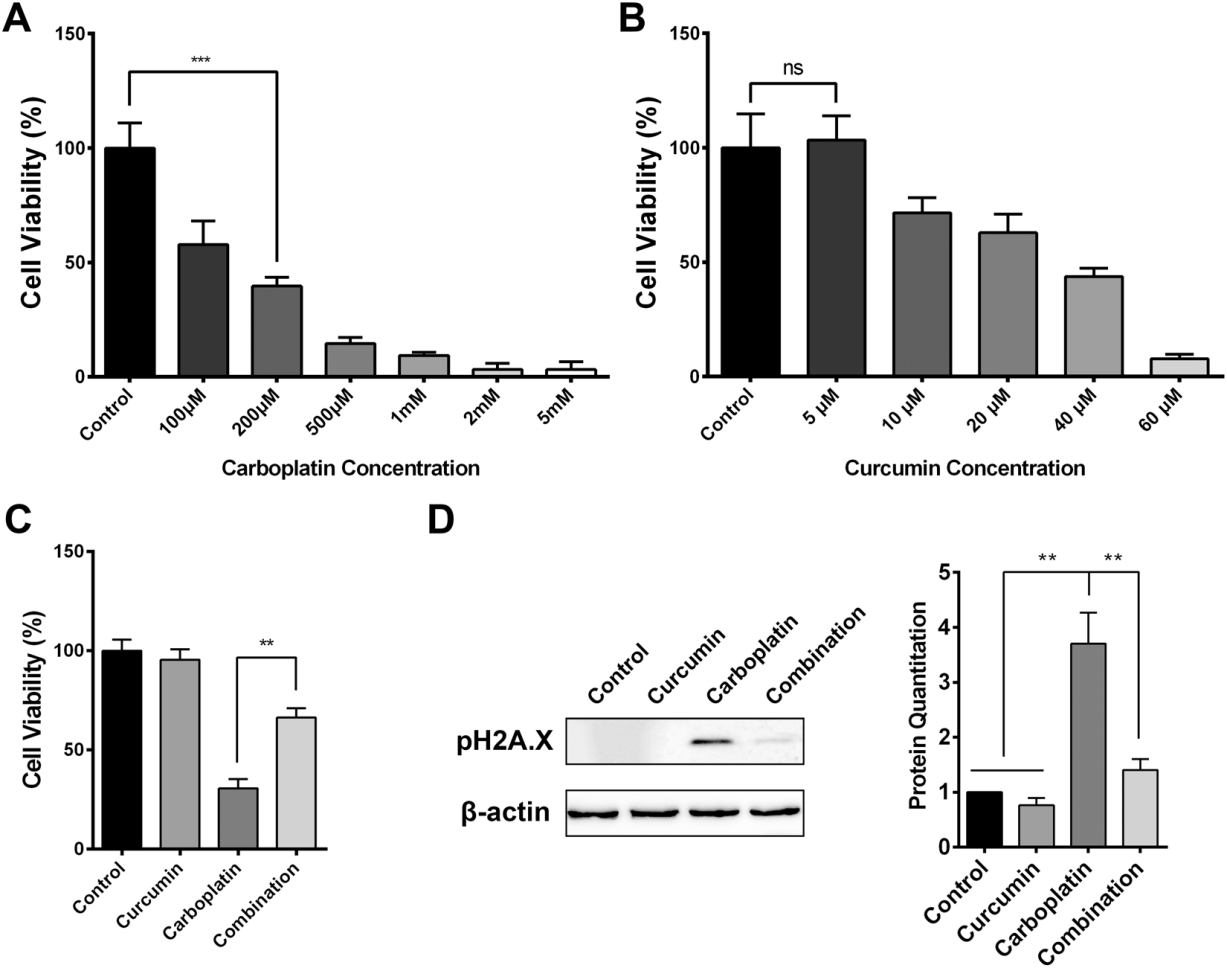
Effect of different concentrations of (**A**) carboplatin and (**B**) curcumin on bone marrow cell viability. (**C**) The attenuated effect of curcumin (5 μM) on carboplatin (200 μM)-induced bone marrow cell damage. (**D**) The expression on pH2A.X in bone marrow cells treated with control, curcumin (5 μM), carboplatin (200 μM) and combination (5 μM curcumin and 200 μM carboplatin). **p < 0.01, ***p < 0.005.

To validate the effects of carboplatin on hematopoietic cells *in vitro*, we next extracted bone marrow cells from tumor-bearing mice and treated them with carboplatin or curcumin. Carboplatin significantly inhibited bone marrow cell viability in a concentration-dependent manner (Fig. 4A). Unlike carboplatin, a low concentration of curcumin did not affect bone marrow cell viability, while a high concentration of curcumin remarkably inhibited bone marrow cell viability (Fig. 4B). Therefore, we take 200 μM for carboplatin and 5 μM for curcumin as our experimental concentration in subsequent assays. The combination results showed that curcumin significantly improved the inhibitory effect of carboplatin on bone marrow cells (Fig. 4C). As platinum-based drugs always cause DNA damage in cancer cells, we hypothesized that carboplatin inhibits bone marrow cell viability through inducing DNA damage. Therefore, we examined the expression of pH2A.X (S139), a DNA damage and mitotic marker^26,27^, in bone marrow cells treated with curcumin or carboplatin. Western blot analysis showed that carboplatin dramatically increased the expression of pH2A.X while treatment with curcumin attenuated the effect (Fig. 4D), indicating that curcumin improves DNA damage induced by carboplatin in bone marrow cells. In conclusion, curcumin attenuates carboplatin-induced DNA damage in bone marrow cells to improve carboplatin-induced myelosuppression.

### Using chemical proteomics to identify CHEK2 and BRCA1 as the targets of curcumin

**Figure 5:**
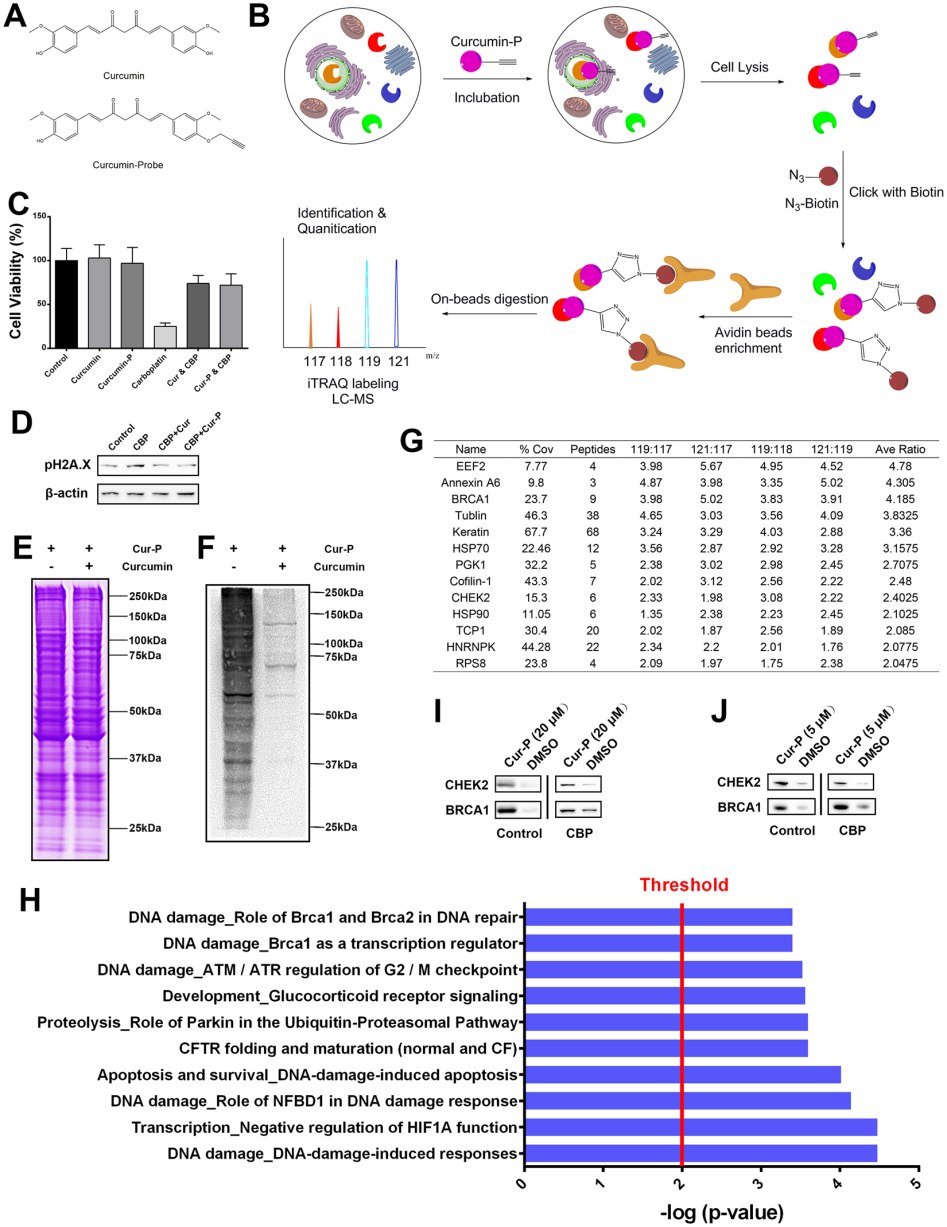
(**A**) Structures of curcumin and the curcumin probe. (**B**) Work flow of target identification in bone marrow cells. (**C**) The attenuated effect of the curcumin probe (5 μM) on carboplatin (200 μM)-induced bone marrow cell damage. (**D**) The attenuated effect of the curcumin probe (5 μM) on carboplatin (200 μM)-induced DNA damage in bone marrow cells. (**E**) Coomassie Brilliant Blue staining of the proteome treated with the curcumin probe (20 μM) alone or the curcumin probe (20 μM) coupled with curcumin (80 μM). (F) ECL of the proteome treated with the curcumin probe alone (20 μM) or the curcumin probe (20 μM) coupled with curcumin (80 μM) followed by the addition of avidin-HRP. (**G**) Confident proteins identified by Cur-P in bone marrow cells (sorted by average enrichment ratios). (**H**) Curcumin-related pathways in bone marrow cells from proteomic analysis. (**I**) Western blot validation of the selected Cur-P (20 μM) targets in bone marrow cells with or without carboplatin treatment. The bolts in different quadrants derived from different gels with different exposures. (**J**) Western blot validation of the selected Cur-P (5 μM) targets in bone marrow cells with or without carboplatin treatment. The bolts in different quadrants derived from different gels with different exposures.

To further elucidate the molecular mechanism involved, we used a chemical proteomics approach to identify the functional targets of curcumin, as illustrated in Fig. 5B. Briefly, a curcumin probe (Cur-P) with an alkyne tag was first designed and synthesized (Fig. 5A). In previous study, Cur-P has been proven to exhibit similar biological activity to curcumin^28^. Cur-P and DMSO (negative control) were subsequently incubated with bone marrow cells to let the target proteins attach to the probe. Cells were then lysed and protein-probe complexes were undergone a “click chemistry” reaction to link with a biotin tag^29^. Next, streptavidin beads were added to enrich the target proteins. The beads were thoroughly washed, and on-beads trypsin digestion was performed. The derived peptides were then reacted with iTRAQ reagents (control samples were labeled with 117 or 118; Cur-P-treated samples were labeled with 119 or 121). Finally, the labeled samples were pooled together and analyzed with LC-MS/MS to identify and quantify the target proteins.

A CCK-8 assay was performed to examine the effect of Cur-P on bone marrow cell viability. Cur-P significantly attenuated the inhibitory effect of carboplatin on cell viability (Fig. 5C). Moreover, similar to curcumin, Cur-P also improved carboplatin-induced DNA damage in bone marrow cells (Fig. 5D). These results indicate that Cur-P exhibits similar biological activity to curcumin. To confirm that Cur-P shares similar targets with curcumin, we performed SDS-PAGE before the addition of streptavidin beads. After Coomassie Brilliant Blue staining, we discovered that the protein abundance of the two groups (Cur-P and curcumin coupled with Cur-P) were highly similar (Fig. 5E). Since the target proteins were attached with a biotin tag through the “click chemistry” reaction, we utilized avidin-HRP to detect the labeled proteins. The probe successfully modified many proteins when used alone, while proteins were rarely labelled after the addition of curcumin due to competitive inhibition (Fig. 5F). These results indicate that Cur-P and curcumin share the same targets in bone marrow cells.

We next utilized iTRAQ method to identify the targets of curcumin in bone marrow cells. For specific protein targets, the iTRAQ reporter ions 119 and 121 have significantly higher intensities than 117 and 118, whereas for non-specific binding and endogenously biotinylated proteins, the reporter intensities are similar. In our study, a total of 134 proteins were successfully identified and quantified. To reduce potential false-positive targets, we set a highly stringent differential ratio of 2 (the average of 119/117, 119/118, 121/117 and 121/118) as the cut-off threshold to differentiate specific binding targets from non-specific targets, which resulted in 13 confident target proteins (Fig. 5G). Then, we used MetaCore^TM^ to thoroughly analysis the identified targets (Fig. 5H) and the results showed that curcumin involved in DNA damage, transcription regulation of HIF1A, apoptosis and survival, CFTR folding and maturation, proteolysis, and glucocorticoid receptor signaling in bone marrow cells. In the case of DNA damage, curcumin is linked with DNA-damage-induced response and cell cycle. Particularly, curcumin involves DNA repair pathway mediated by BRCA1 and BRCA2. In other cases, HIF1A functions as a master regulator of cellular and systemic homeostatic response to hypoxia by activating transcription of many genes, including those involved in energy metabolism, angiogenesis, apoptosis, and other genes whose protein products increase oxygen delivery or facilitate metabolic adaptation to hypoxia^30^; CFTR functions as a channel across the membrane of cells that produce mucus, sweat, saliva, tears, and digestive enzymes^31^; and glucocorticoids are steroid hormones regulated in a circadian and stress-associated manner to maintain various metabolic and homeostatic functions that are necessary for life^32^. Among them, DNA damage repair is closely related to our findings above. Among the target proteins, BRCA1 and CHEK2 are involved in DNA repair pathway. Western blot analysis of the pull-down samples was conducted, and BRCA1 and CHEK2 were validated as the targets of curcumin in bone marrow cells (Fig. 5I). In addition, we also utilized curcumin probe to pull down target proteins in carboplatin-treated bone marrow cells, and results showed that BRCA1 and CHEK2 were still the protein targets of curcumin (Fig. 5I). As we take 5 μM as curcumin’s concentration in the study to attenuate carboplatin induced toxicity, we also used 5 μM curcumin probe to pull down proteins in bone marrow cells with or without carboplatin treatment, and results suggested that 5 μM curcumin probe could also pull down more proteins of CHEK2 and BRCA1 than that of control (Fig. 5J). Collectively, CHE2 and BRCA1 are the protein targets of curcumin in bone marrow cells.

### Curcumin attenuates DNA damage induced by carboplatin by up-regulating the expression of ERCC1, BRCA1 and BRCA2

**Figure 6:**
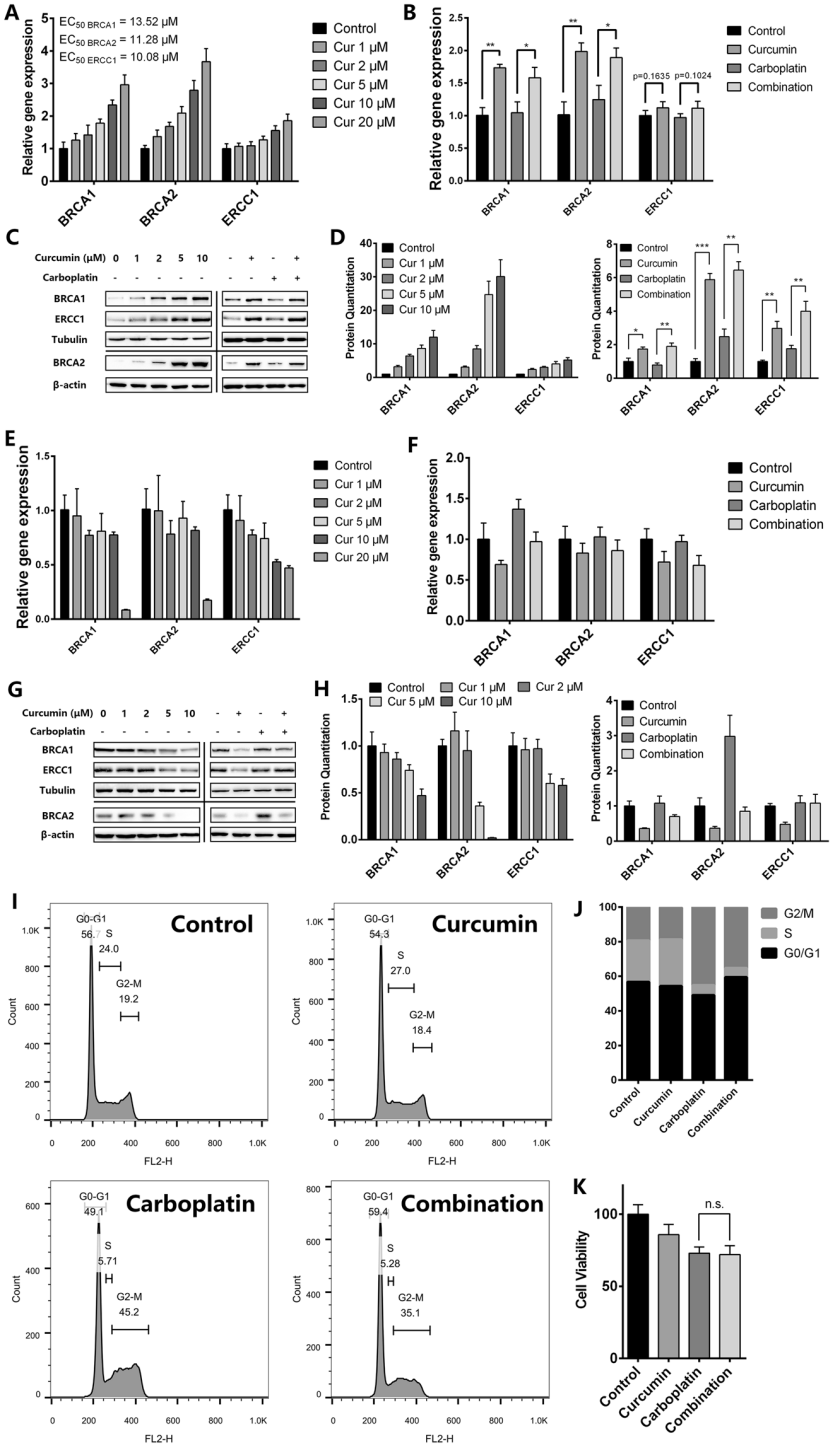
qPCR was used to examine the mRNA expression of BRCA1, BRCA2 and ERCC1 in (**A**,**B**) bone marrow cells and (E & F) T241 cells treated with different concentrations of curcumin and control, curcumin (5 μM), carboplatin (200 μM) and combination (5 μM curcumin and 200 μM carboplatin). Western blot revealed the protein expression of BRCA1, BRCA2 and ERCC1 in (**C**,**D**) bone marrow cells and (**G**,**H**) T241 cells treated with different concentrations of curcumin and control, curcumin (5 μM), carboplatin (200 μM) and combination (5 μM curcumin and 200 μM carboplatin). The bolts in different quadrants derived from different gels with different exposures. (**I**,**J**) Cell cycle analysis of T241 cells treated with control, curcumin (5 μM), carboplatin (200 μM) and combination (5 μM curcumin and 200 μM carboplatin). (**K**) Cell viability of T241 cells treated with control, curcumin (5 μM), carboplatin (200 μM) and combination (5 μM curcumin and 200 μM carboplatin). *p < 0.05, **p < 0.01, ***p < 0.005.

Since curcumin involves in DNA repair pathway in bone marrow cells, we inferred that curcumin might improve carboplatin-induced DNA damage by activating the DNA repair pathway. Previous reports showed that the DNA lesions caused by platinum salts were repaired by a combination of nucleotide excision repair (NER) and homologous recombination^33^. As BRCA1, BRCA2 and ERCC1 play important roles in the process of NER and homologous recombination^34,35^, qPCR analysis was conducted to examine the expression of BRCA1, BRCA2 and ERCC1 in bone marrow cells following treatment with curcumin or carboplatin. Curcumin increased the expression of BRCA1, BRCA2 and ERCC1 in a concentration-dependent manner (Fig. 6A). Consistently, 5 μM curcumin increased the expression of BRCA1 and BRCA2 significantly in carboplatin-treated bone marrow cells, and up-regulated ERCC1 expression to some extent (Fig. 6B). Results from western blot analysis reinforced the conclusion (Fig. 6C and D). The results reveal that curcumin activates NER and homologous recombination in bone marrow cells through up-regulating BRCA1, BRCA2 and ERCC1 expression. To sum up, curcumin attenuated DNA damage in bone marrow cells induced by carboplatin by activating the DNA repair pathway.

Considering that curcumin increases the carboplatin resistance of bone marrow cells, it is alarming that curcumin might also induce carboplatin resistance in tumor cells, thereby reducing the therapeutic effect of carboplatin. Therefore, we examined the effect of curcumin on DNA repair pathways in tumor cells. Results from qPCR and western blot analyses demonstrated that curcumin did not increase the expression of BRCA1, BRCA2 and ERCC1 in T241 cells or carboplatin-treated T241 cells (Fig. 6E–H), indicating that curcumin does not activate the DNA repair pathway in T241 cells. As DNA damage leads to G2/M cell cycle arrest^36^, we next examined the effects of curcumin and carboplatin on the cell cycle in T241 cells by flow cytometry. Carboplatin induced G2/M cell cycle arrest, whereas curcumin had no obvious effect (Fig. 6I and J). Moreover, curcumin had no effect on the inhibition of T241 cell viability induced by carboplatin (Fig. 6K). From these results, we conclude that curcumin does not increase the carboplatin resistance of T241 cells.

## Discussion

As myelosuppression weakens the therapeutic effects of carboplatin (i.e., prolonging the life span of tumor-bearing patients, to some extent), attenuating the side effects of carboplatin has become a hot therapeutic target to treat tumor-bearing patients. Zhang *et al*. reported that anti-VEGF therapy improved survival by reducing carboplatin-induced chemotoxicity^37^. Together with our previous finding that curcumin suppressed VEGF production from tumor cells and inhibited the VEGF-VEGFR2 signaling pathway^20^, we inferred that the combination of curcumin and carboplatin may result in an improved life span compared to treatment with carboplatin alone. As carboplatin and curcumin both exhibit anti-cancer activity^38,39^, we first examined the synergistic effect of the two agents on tumor growth; however, there was no obvious synergistic effect. Based on survival rate analysis, curcumin remarkably improved the survival rate of tumor-bearing mice treated with carboplatin. The bone marrow and kidney from tumor-bearing mice were histologically examined, which confirmed that curcumin attenuated the side effects of carboplatin by improving myelosuppression. To elucidate the mechanism involved, we used a chemical proteomics approach to identify the targets of curcumin in bone marrow cells; CHEK2 and BRCA1 were identified as the protein targets of curcumin, which indicates that the attenuated effect of curcumin is related to DNA repair. Western blot and qPCR were utilized to detect the effect of curcumin on the expression of BRCA1, BRCA2 and ERCC1, which are key proteins involved in the DNA repair process. Curcumin activated NER and homologous recombination by up-regulating BRCA1, BRCA2 and ERCC1 expression, thereby improving carboplatin-induced myelosuppression to extend the life span of mice.

The results that curcumin attenuates carboplatin-induced chemotoxicity by improving carboplatin-induced myelosuppression are in agreement with previous reports. Zhang *et al*. reported that tumor-derived VEGF impairs bone marrow hematopoiesis, shortens survival and increases chemotoxicity and chemotherapy-induced mortality^18^. They also discovered that the anti-VEGF therapeutic sunitinib improves survival by reducing carboplatin-induced chemotoxicity. Of interest, our previous study showed that curcumin blocks VEGFR2 activation and its downstream signaling proteins and suppresses VEGF secretion from tumor cells both *in vitro* and *in vivo* ^20^. In the present study, we demonstrated that carboplatin leads to serious myelosuppression and curcumin, as an anti-VEGF agent, attenuates the effect to some extent.

In addition to the anti-VEGF activity of curcumin described above, we identified another novel mechanism involved in the attenuated effect of curcumin using a chemical proteomics approach. There is no doubt that identifying the direct targets is the most reliable and direct method to determine the mechanism of action of small molecules. Chemical proteomics has been widely applied for target identification of natural products, drugs and small molecules^40,41^. Here, a probe derived from curcumin was utilized to screen the protein targets of curcumin in bone marrow cells. In contrast to natural curcumin, the curcumin probe is attached with an alkyne tag. The tag has nearly no effect on the biological activity of curcumin due to its small size. Wang *et al*. treated HCT116 colon cancer cells with curcumin or curcumin probe in parallel in the functional assays, including ROS, Lysotracker staining and autophagy flux assay, and no significant differences were observed between the drug and the probe^28^, which indicated that the two compounds exhibit similar bioactivities. Based on the conclusions above and our curcumin-competitive inhibition experiment, the curcumin probe is believed to share the same targets with the parent curcumin.

As CHEK2 and BRCA1 were identified as target of the curcumin probe, we can infer that they are also targets of curcumin in bone marrow cells. CHEK2 operates in a complex network of proteins to elicit DNA repair, cell cycle arrest or apoptosis in response to DNA damage^42^, and BRCA1 controls homology-directed DNA repair^43^. Considering that carboplatin treatment leads to DNA damage in bone marrow cells, we next examined the effect of curcumin on DNA repair, including the expression of BRCA1, BRCA2 and ERCC1. ERCC1 plays a key role in NER, and reduced levels of ERCC1 lead to a deficiency in NER^44,45^. BRCA1 and BRCA2 mediate homologous recombination in DNA repair and inhibition of the BRCA pathway can enhance cisplatin sensitivity in cancer cell lines^46^. Our results showed that curcumin significantly increased the expression of BRCA1, BRCA2 and ERCC1, which indicates that curcumin improves myelosuppression by activating NER and homologous recombination.

In the case of bone marrow cells, carboplatin resistance results in the attenuated effect on myelosuppression induced by carboplatin but weakens the therapeutic effect of carboplatin on cancer cells^47^. Therefore, we also examined the effect of curcumin on the DNA repair pathway in T241 tumor cells. Curcumin down-regulated the expression of BRCA1, BRCA2 and ERCC1 in T241 cells, suggesting that curcumin enhances the sensitivity of T241 cells to carboplatin treatment. Wang *et al*. reported that curcumin-enhanced chemosensitivity of FDA-approved platinum (II)-based anti-cancer drugs involves the down-regulation of nuclear endonuclease G and NF-κB, as well as the induction of apoptosis and G2/M arrest^48^, which is consistent with our results. As a result, curcumin leads to carboplatin resistance in bone marrow cells to improve myelosuppression induced by carboplatin and enhances the sensitivity of T241 tumor cells to carboplatin treatment to some extent.

However, considering BRCA1 and BRCA2 were thought be tumor suppressor proteins, it is alarming that the inhibitory effect of curcumin on the expression of BRCA1 and BRCA2 in T241 cells might lead to tumor progression. BRCA1 and BRCA2 play important roles in maintaining genomic integrity by protecting cells from double-strand breaks (DSB) that arise during DNA replication or after DNA damage, namely the two proteins prevent gene mutation to suppress tumorigenesis^49^. In our study, carboplatin inhibits tumor cells through inducing DNA damage, and curcumin-induced decreases in BRCA1 and BRCA2 expression contribute to carboplatin’s cytotoxic effect. Therefore, they are not contradictory. In addition, some reports have shown that curcumin inhibits BRCA1 expression to suppress DNA repair pathway, so as to inhibit cell viability and increase sensitivity against cisplatin. For example, Ting *et al*. reported that curcumin triggered DNA damage and reduced BRCA1 expression to suppress DNA repair pathway in human lung cancer cells, thereby inhibiting cell viability^50^. Lu *et al*. showed that curcumin induced DNA damage and inhibited DNA repair pathway through decreasing BRCA1 expression in mouse-rat hybrid retina ganglion cells (N18)^51^.

In conclusion, our findings suggest that the conbination therapy of curcumin and carboplatin improves myelosuppression induced by carboplatin, increases the survival rate of tumor-bearing mice and provides novel mechanistic insight to explain the benefits of curcumin with respect to carboplatin-induced side effects. As curcumin is a safe food supplement, our study provides evidence that curcumin can be further developed as a potential combination agent with carboplatin in the future.

## Material and Methods

### General information

Curcumin, Dimethyl sulfoxide (DMSO), streptavidin beads, methanol, urea, Tris (2-carboxyethyl) phosphine (TCEP) phosphoric acid, tris [(1-benzyl-1H-1,2,3-triazol-4-yl) methyl]amine (TBTA) and CuSO_4_ were obtained from Sigma-Aldrich (St. Louis, MO). Methyl methanethiosulfonate (MMTS) was purchased from Pierce (Rockford, IL, USA). Biotin-azide were obtained from Click Chemistry Tools (Scottsdale, AZ, USA). The antibodies used included: β-actin (A5441) from Sigma Aldrich (St. Louis, MO, USA), pH2A.X (#2577 S), CHEK2 (#2662), ERCC1 (#3885), Tubulin (#3873) from Cell Signaling Technology (Danvers, MA, USA), and BRCA1 (ab191042), BRCA2 (ab27976) from Abcam (Cambridge, UK). All methods were performed in accordance with the relevant guidelines and regulations.

### Cell culture

The bone marrow cells were maintained in tissue culture using RPMI medium 1640 (HyClone, Logan, UT, USA) supplemented with 10% fetal calf serum (HyClone, Logan, UT, USA) and 1% penicillin-streptomycin (Invitrogen, Carlsbad, CA, USA). All cells were cultured in a humidified CO_2_ incubator at 37 °C.

### Animals

All animal experiments were carried out in accordance with the protocol approved by Nanjing University Animal Care and Use Committee (20130118).

### Bone marrow cell isolation

Femur from mouse was soaked with 75% ethanol for 3 min. Wash the femur with PBS and then cut both ends of the femur. Use PBS to swash the bone marrow into an Eppendorf tube. Centrifuge at 400 g for 3 min and remove the supernatant. The bone marrow was incubated with ACK Lysis Buffer (Thermofisher; A1049201) for 1 min. Centrifuge at 400 g for 3 min and remove the supernatant again. Wash the marrow with PBS twice.

### Cell viability assay

5 × 10^3^ cells in 50 μL medium per well were seeded on 96 well plate and incubated at 37 °C overnight, then treated with 50 μL various doses of compounds for 48 h. 10 μL CCK-8 solution per well was added and incubated for 4 h at 37 °C. Absorbance at 650 nm was measured.

### Western blotting analysis

Total proteins were extracted from cells and separated using 10% SDS-PAGE and then electrophoretically transferred to a nitrocellulose membrane (Millipore; HATF00010). The membrane was blocked with 5% milk in PBST and incubated with primary antibody and secondary antibody. Target proteins were detected with ECL detection reagent (Thermo Scientific; 34075).

### Blood sample analysis

Animal blood was collected by eyeball removal in the presence of anticoagulant EDTA. Hematological parameters including white blood cells, red blood cells, and platelet counts were measured by haematology analyser (Sysmex XT-2000iv, Sysmex, Japan).

### Histological studies

Paraffin-embedded tissues were sectioned in 4 μm thickness and stained with hematoxylin-eosn (H&E) according to previously described methods^52^.

### *In situ* labeling of bone marrow cells by Cur-P

Bone marrow cells were maintained in 150 mm dishes. The cells were incubated with Cur-P or DMSO respectively at 37 °C for 4 h. The cells were washed twice with PBS after removal of the media. The cells were lysed with 20 mM Tris (pH 7.5), 150 mM NaCl and 1% Triton X-100. The cell lysate was centrifuged at 10000 rpm for 45 minutes at 4 °C to remove debris and insoluble fraction. Protein concentration of the lysates was determined with BCA protein quantitation kit. Equal amount of proteins (4 mg) were incubated with 10 μM biotin-azide, 1 mM TCEP, 100 μM TBTA and 1 mM CuSO_4_ for 4 h at room temperature to attach the target proteins with biotin tag. The labeled proteins were then acetone-precipitated and air-dried. The samples were then solubilized with 100 μL of 1× SDS loading buffer and 15 μL of each samples were loaded into wells of 10% polyacrylamide gel and separate through SDS gel electrophoresis. Coomassie Brilliant Blue Staining was applied to visualize the total proteins. After SDS-PAGE, proteins were electrophoretically transferred to a nitrocellulose membrane, followed by avidin-HRP incubation. ECL luminescence was applied to visualized the modified proteins.

### Curcumin target identification using quantitative chemical proteomics

Bone marrow cells were maintained in 150 mm dishes. The cells were incubated with 20 μM Cur-P or DMSO respectively at 37 °C for 4 h. The cells were washed twice with PBS after removal of the media. The cells were lysed with 20 mM Tris (pH 7.5), 150 mM NaCl and 1% Triton X-100. The cell lysate was centrifuged at 10000 rpm for 45 minutes at 4 °C to remove debris and insoluble fraction. Protein concentration of the lysates was determined with BCA protein quantitation kit. Equal amount of proteins (4 mg) were incubated with 10 μM biotin-azide, 1 mM TCEP, 100 μM TBTA and 1 mM CuSO_4_ for 4 h at room temperature to attach the target proteins with biotin tag. The labeled proteins were then acetone-precipitated and air-dried. The samples were then solubilized with 700 μL PBS containing 1.2% SDS. The samples were then treated with streptavidin beads for 4 h at room temperature to enrich the labeling proteins. The beads were washed with PBS (1% SDS), PBS (0.1% SDS), 6 M Urea, PBS and double distilled water several times to remove non-specific binding proteins. After on-beads digestion, the target proteins were identified with iTRAQ according to the previously published method^28^.

### Statistical analysis

GraphPad prism 5.0 was used for statistical analysis. Data was summarized as mean ± SEM. One way ANOVA was used to determine the significant differences between groups. Results were considered to be significant for p-values of < 0.05.

## Electronic supplementary material

Supplementary Information
