# Supplementary material for: Curcumin activates DNA repair pathway in bone marrow to improve carboplatin-induced myelosuppression
Source: Sci Rep. 2017 Dec 18;7:17724. doi: 10.1038/s41598-017-16436-9 (PMC5735145; doi:10.1038/s41598-017-16436-9)
Supplement: Supplementary file 1 — Supplementary Information [file 41598_2017_16436_MOESM1_ESM.pdf]

Supplementary Information for:

Curcumin activates DNA repair pathway in bone marrow to improve carboplatin-induced myelosuppression

Xiao Chen[1,+], Jigang Wang[1,3,+], Zhongping Fu[2], Bo Zhu[1], Jie Wang[1], Shengwen Guan[1], Zichun Hua[1,4,5,*]

[1]The State Key Laboratory of Pharmaceutical Biotechnology, School of Life Sciences, Nanjing University, Nanjing, 210023, China
[2]State Key Laboratory of Quality Research in Chinese Medicines, Macau University of Science and Technology, Avenida Wai Long, Taipa, Macau
[3]Department of Biological Science, National University of Singapore, Singapore, 117543, Singapore
[4]Changzhou High-Tech Research Institute of Nanjing University and Jiangsu TargetPharma Laboratories Inc., Changzhou, 213164, China
[5]Shenzhen Research Institute of Nanjing University, Shenzhen, 518057, China
[*]Correspondence: zchua@nju.edu.cn
[+]These authors contributed equally to this work

This supplement contains:

Supplementary Material and Methods

MetaCore™ analysis. The target proteins of curcumin identified with iTRAQ were analyzed with MetaCore™ network building tool (GeneGo Inc., St. Joseph, MI) to identify the possible pathways involved. MetaCore™ is formed by an annotated database of protein interactions and metabolic reactions. The databases consist of millions of relationships between proteins that are derived from literature publications on proteins and small molecules. The Uniprot ID of the identified genes were imported into MetaCore™. Hypothetical networks of proteins from our experiment were then built using the shortest paths algorithm, one of the several algorithms integrated within MetaCore. The results (the maps with the list of proteins from the uploaded data set) were then compared with all the possible pathway maps for all the proteins in the database, and the *p* value (the probability of randomly obtaining an intersection of a certain size between the uploaded proteins and the proteins on the pathway maps follows a hypergeometric distribution) was calculated based on hypergeometric distribution probability test. The relevant pathway maps were then prioritized based on their statistical significance with respect to the uploaded data sets.
